# Limb Differences in Unipedal Balance Performance in Young Male Soccer Players with Different Ages

**DOI:** 10.3390/sports7010020

**Published:** 2019-01-11

**Authors:** Thomas Muehlbauer, Gerrit Schwiertz, Dennis Brueckner, Rainer Kiss, Stefan Panzer

**Affiliations:** 1 Division of Movement and Training Sciences/Biomechanics of Sport, University of Duisburg-Essen, 45141 Essen, Germany; g.schwiertz@hotmail.de (G.S.); dennis.brueckner@uni-due.de (D.B.); 2 Department of Health and Social Affairs, FHM Bielefeld—University of Applied Sciences, 33602 Bielefeld, Germany; kiss@fh-mittelstand.de; 3 Institute of Sport Science, Saarland University, 66123 Saarbrücken, Germany; s.panzer@mx.uni-saarland.de

**Keywords:** athletes, postural control, one-legged balance performance, Lower Quarter Y Balance Test

## Abstract

In soccer, the dominant leg is frequently used for passing and kicking while standing on the non-dominant leg. Consequently, postural control in the standing leg might be superior compared to the kicking leg and is further enhanced with increasing age (i.e., level of playing experience). Unfortunately, leg differences in postural control are associated with an increased risk of injuries. Thus, we examined differences between limbs in unipedal balance performance in young soccer players at different ages. Performance in the Lower Quarter Y Balance Test (YBT-LQ) of the dominant and non-dominant leg and anthropometry was assessed in 76 young male soccer players (under-13 years [U13]: *n* = 19, U15: *n* = 14, U17: *n* = 21, U19: *n* = 22). Maximal reach distances (% leg length) and the composite scores were used for further analyses. Statistical analyses yielded no statistically significant main effects of leg or significant Leg × Age interactions, irrespective of the measure investigated. However, limb differences in the anterior reach direction were above the proposed cut-off value of >4 cm, which is indicative of increased injury risk. Further, statistically significant main effects of age were found for all investigated parameters, indicating larger reach distances in older (U19) compared to younger (U13) players (except for U15 players). Although reach differences between legs were non-significant, the value in the anterior reach direction was higher than the cut-off value of >4 cm in all age groups. This is indicative of an increased injury risk, and thus injury prevention programs should be part of the training of young soccer players.

## 1. Introduction

During soccer practice and games, passing and kicking are the most frequently used playing techniques, preferably performed with the dominant leg while the non-dominant leg is used as standing leg [1]. As a consequence, postural control in the standing leg might be superior compared to the kicking leg. This leg difference in unipedal balance performance may be further enhanced based on athletes’ soccer experience (i.e., years of soccer training). In this regard, Paillard [2] reported that long-term motor practice induced structural and functional adaptations in the postural control system. That is, adaptations in the contribution of sensory pathways (e.g., less dependency on visual information), central structures (e.g., reduced reflex activities), and motor functions (e.g., decreased muscle coactivation). Further, he stated that the induced adaptations are specific to the context in which the postural task is practiced. According to the motor practice regimens of soccer players, the predominant use of the dominant leg for passing and kicking and the non-dominant leg for standing may result in limb differences in unipedal balance performance.

Previous studies investigating leg-based postural control in soccer players at different ages (i.e., levels of experience) showed inconsistent findings [3,4,5,6]. Specifically, some studies [3,4] did not detect significant differences between age groups while others [5,6] revealed significant differences in unipedal balance performance in older, more experienced—compared to younger, less experienced—athletes. For example, Butler et al. [3] investigated leg differences in maximal reach distances in the Lower Quarter Y Balance Test (YBT-LQ) using soccer players playing at high school, collegiate or professional level. They reported no significant limb differences on the YBT-LQ among groups in any of the three reach directions. In contrast, Ricotti et al. [5] examined postural sway while performing the one-legged stance in Italian soccer players from different leagues. Results showed significantly pronounced balance performance for the standing compared to the kicking leg in more (e.g., 2nd league) compared to less (e.g., 3rd league) experienced players. The authors attributed these findings to the more frequent use of the standing leg to stabilize the body during soccer-related movements (e.g., passing and/or kicking). Further, Breen and colleagues [6] argued that motor control strategies might differ between older, more experienced players and younger, less experienced players.

From a health perspective, reach distance differences between legs in the YBT-LQ can be seen as a risk factor for sustaining injuries of the lower extremities [7,8]. For example, Plisky et al. [7] measured YBT-LQ performance and documented the incidence of lower limb injuries in high school basketball players. Results showed that 54 out of 235 players were injured during one season, and particular players with an anterior reach distance difference of more than 4 cm between the left and right leg were 2.5 times more likely to sustain ankle sprains and anterior cruciate ligament (ACL) tears. In addition, Smith et al. [8] tested YBT-LQ performance in college athletes and registered the number of lower limb injuries throughout one season. Eighty-one out of 184 athletes were injured. Further, they reported that athletes with an anterior left/right leg reach difference of more than 4 cm showed a 2.2 times increased risk of sustaining a lower limb injury.

Thus, the purpose of the present study was to investigate limb differences in unipedal balance performance in young male soccer players across several age groups. We expected that age (i.e., level of playing experience) would positively affect YBT-LQ performance, with older, more experienced players showing greater reach distances than younger, less experienced players. Further, we assumed larger reach distance differences between the standing leg and kicking leg in the YBT-LQ for older compared to younger soccer players, due to the task-specific structural and functional adaptations in the postural control system that occur because of the longer exposure of their standing leg for stabilization purposes while performing soccer-related actions with their kicking leg.

## 2. Materials and Methods

### 2.1. Participants

Seventy-six male sub-elite young soccer players participated in this cross-sectional study after experimental procedures were explained (Table 1). Participants played in the highest or second highest league of their respective age group. Training experience ranged between 7.1 ± 1.0 years (U13) and 10.2 ± 1.9 years (U19), and training volume ranged between 270 min/week (U13) and 450 min/week (U19). None of the participants reported a history of musculoskeletal, neurological or orthopedic disorders or injuries. Participants’ approval and parents’ informed consent was obtained prior to the study start, and the study protocol was approved by The Human Ethics Committee at the University of Duisburg-Essen, Germany (TM_27.2.2018).

### 2.2. Procedures

Upon entering the testing room, the dominant leg (i.e., the preferential use of one leg over the other to perform a dynamic action) was determined using the following question: “Which foot do you use to kick a ball?” Subsequently, the testing procedure (i.e., measurement of anthropometric variables followed by performance assessment in the YBT-LQ) was explained using standardized verbal instructions and a visual demonstration.

### 2.3. Anthropometry

Participants’ body height was registered barefoot to the nearest 0.5 cm using a stadiometer (seca 217, seca®, Basel, Switzerland). Body mass was determined to the nearest 100 g with an electronic scale (seca 803, seca®, Basel, Switzerland), and the body mass index (BMI; body mass divided by height squared (kg/m^2^) was calculated. Participants’ length of the kicking (dominant) leg and standing (non-dominant) leg was assessed in supine position and defined as distance from the anterior superior iliac spine to the most distal aspect of the medial malleolus [7].

### 2.4. Lower Quarter Y Balance Test

**Figure 1:**
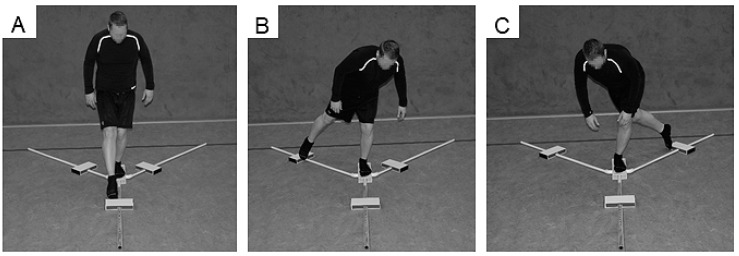
Participant performing the Lower Quarter Y-Balance Test (YBT-LQ) anterior (ANT) (**A**), posteromedial (PM) (**B**), and posterolateral (PL) (**C**) reach.

The Y Balance Test Kit (Functional Movement Systems, Chatham, MA, USA) was used to register YBT-LQ performance (Figure 1). Participants were asked to reach with one leg as far as possible in anterior (ANT), posteromedial (PM), and posterolateral (PL) directions while standing on a centralized stance platform and maintaining balance. The YBT-LQ was performed without shoes with the standing leg and kicking leg. Players were instructed to conduct three practice and three continuously performed data-collection trials. In order to prevent effects of fatigue, participants rested for one minute between trials. As stated by Plisky [9], players started with the right leg placed behind the red starting line of the centralized stance plate and the left leg touching and moving the reach indicator with the most distal part of the foot. Subsequently, participants returned to a bipedal stance position. The test procedure was as follows: (1) Standing on the right leg and reaching with the left leg in the ANT direction and vice versa, (2) standing on the right leg and reaching with the left leg in the PM direction and vice versa, and (3) standing on the right leg and reaching with the left leg in the PL direction and vice versa [9]. An examiner documented the distance (i.e., from the center of the centralized stance platform to the maximal reach distance) after each reach. Trials were discarded and repeated once if the player (1) lost his balance (i.e., stepped with the reach leg on the ground) at any point during the trial, (2) lifted the heel of the stance leg from the centralized stance platform, (3) stepped on top of the reach indicator for support, or (4) kicked the reach indicator. The examiner was trained to guide and instruct the YBT-LQ protocol. The YBT-LQ is a valid and reliable test for the assessment of unipedal balance performance in youth and young adults [10,11,12]. Further, low values were reported for the standard error of measurement, indicating that the YBT-LQ has an adequate level of accuracy [11]. Reach distances in each of the three directions (i.e., ANT, PM, PL) were normalized (%) to the left and right leg length (LL), respectively, and used as outcome measure. The formula for the calculation is: Normalized maximal reach distance (% leg length [LL]) = (maximal reach distance [cm])/LL [cm]) × 100. Further, the normalized (% LL) composite score (CS) was calculated for each leg using the following formula provided by Filipa et al. [13]: CS = ((ANT + PM + PL)/(LL × 3)) × 100. In addition, the non-normalized reach difference (cm) between the standing leg and kicking leg for each of the three directions was calculated and averaged to assess leg asymmetry. For the ANT direction, a reach difference between legs of >4 cm is associated with an increased risk of sustaining a non-contact lower extremity injury (e.g., ankle sprains, ACL tears) [7,8].

### 2.5. Statistical Analysis

Data are presented as group mean values ± standard deviations (SD). Normal distribution was examined using Kolmogorov–Smirnov tests. Subsequently, a 4 (age: U13, U15, U17, U19) × 2 (leg: dominant leg, non-dominant leg) analysis of variance (ANOVA) with repeated measures on leg was performed to analyze YBT-LQ performance. Post-hoc tests with Bonferroni-adjusted α were used to identify comparisons that were statistically significant. Effect sizes (*f*), determined from partial eta-squared (*η_p_*^2^), were classified as small (0 ≤ *f* ≤ 0.24), medium (0.25 ≤ *f* ≤ 0.39), and large (*f* ≥ 0.40) [14]. All analyses were performed using the Statistical Package for Social Sciences (SPSS) version 24.0, and the significance level was set at *p* < 0.05.

## 3. Results

**Figure 2:**
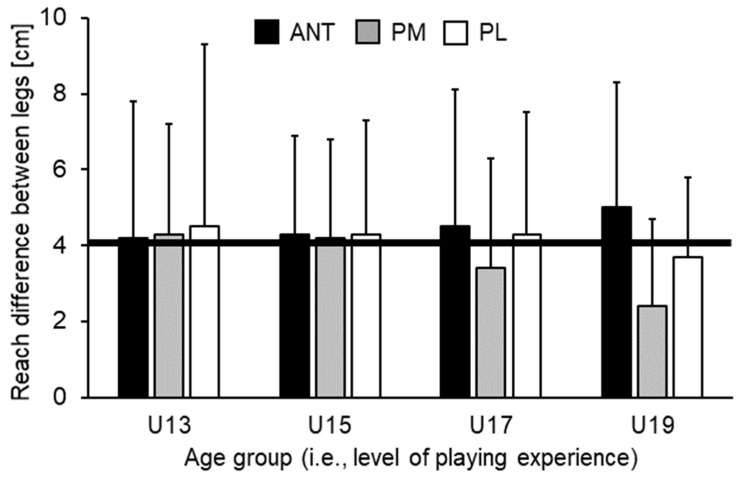
Reach distance differences (cm) between kicking (dominant) and standing (non-dominant) leg in the ANT (anterior), PM (posteromedial), and PL (posterolateral) reach directions by age group. The black solid line corresponds to reach distance differences between the standing and kicking leg of 4 cm. For the ANT direction, a reach distance difference greater than 4 cm is related to an increased risk of sustaining a lower extremity injury [7,8]. U13 = under-13 years etc.

Table 2 illustrates means, SDs, and the corresponding ANOVA outcomes for the normalized YBT-LQ performance. Analysis of variance did not show statistically significant main effects of leg (all *p* > 0.05) nor statistically significant Leg × Age interactions (all *p* > 0.05) for any of the investigated measures. However, statistically significant main effects of age were found for each direction and score (all *p* < 0.05, *f* = 0.36–0.59). For ANT (*p* = 0.030) and PM (*p* = 0.033) direction, post-hoc analysis indicated that U15 players achieved significantly shorter reach distances compared to U19 players. No other comparison reached the level of statistical significance. Regarding PL direction and the CS, post-hoc analysis indicated that U15 players reached significantly shorter distances compared to U13 (PL: *p* < 0.001; CS: *p* = 0.005) and U19 players (PL: *p* < 0.001; CS: *p* < 0.001). Further, a tendency towards significantly shorter reach distances (PL: *p* = 0.051) in U15 compared to U17 players could be found. Figure 2 shows non-normalized differences in reach distances between legs across age groups. Irrespective of age, leg differences for the ANT direction were above the proposed cut-off value of >4 cm and increased with age (U13 = 4.2 ± 3.6 cm, U15 = 4.3 ± 2.6 cm, U17 = 4.5 ± 3.6 cm, U19 = 5.0 ± 3.3 cm). Further, limb difference decreased for the PM and PL directions with age but did not reach the level of statistical significance. When looking at the individual data, we additionally found that 7 (37%) out of 19 (U13), 6 (43%) out of 14 (U15), 10 (48%) out of 21 (U17), and 12 (55%) out of 22 (U19) players achieved limb differences for the ANT direction that were over the 4 cm threshold.

## 4. Discussion

The present study investigated limb differences in unipedal postural control in 76 male sub-elite young soccer players at different ages (i.e., level of playing experience). We compared YBT-LQ performance for the standing (non-dominant) leg and kicking (dominant) leg between U13, U15, U17, and U19 players and calculated reach distance differences between legs. Results indicated that: (i) Older, more experienced players (U19) showed significantly greater reach distances than younger, less experienced players (U13) in the majority of comparisons, (ii) irrespective of age, no statistically significant leg differences in unipedal balance performance was found, and (iii) reach differences between the limbs in the ANT direction was above the proposed YBT-LQ cut-off value of >4 cm [7,8] in all four groups and increased with age.

The finding of a better unipedal balance performance (i.e., greater maximal reach distances) in older, more experienced compared to younger, less experienced soccer players is in accordance with our first hypothesis. It is also partly in line with the literature regarding differences in postural control across performance levels within soccer. Using the YBT-LQ, Butler et al. [3] for instance showed greater reach distances and a larger CS in soccer players playing at professional and collegiate compared to high-school level. Further, Ricotti et al. [5] reported less postural sway for the single leg stance in professional compared to non-professional soccer players. Lastly, Paillard and colleagues [15] found better balance performances (i.e., less postural sway) for the single leg stance in soccer players playing at national compared to regional level. This may be a result of older, more experienced players being exposed to higher amounts of training/competition exposition in balance exercises and balance-demanding situations. In addition, differences in physical, coordinative, and proprioceptive aspects between players at different levels might also explain the mentioned findings, because more experienced compared to less experienced athletes are physically stronger, show less movement variability and faster reaction times [5]. Further, the observed differences may be explained by structural and functional adaptations in the postural control system induced by motor experience [2]. Concerning the PL direction and the CS, U15 compared to U13 players showed inferior balance performances. This unexpected finding can be related to the inconsistent growth of lower and upper body parts as well as to maturational changes during adolescence [16].

We further assumed significantly larger differences in reach distances between legs in the YBT-LQ for more experienced compared to less experienced soccer players. More experienced players show a more frequent use of the standing leg in order to stabilize body posture during most soccer-related movements (e.g., passing, kicking). Contrary to our second hypothesis, we detected no statistically significant differences in reach distances between legs among age groups. This finding is in line with Butler et al. [3] and Mala et al. [4] but contrary to those yielded by Ricotti et al. [5] and Breen et al. [6]. A common explanation for this finding is that, although passing and kicking are often-used techniques in soccer which are predominantly performed with the dominant leg and the non-dominant leg used as standing leg [1], there are many situations during training and competition that are performed with both legs. For example, soccer drills like short sprints, change of direction speed exercises, and jump/landing tasks represent important demands during training and competition. In addition, modern training regimens are characterized by the bilateral execution of exercises during soccer [17]. Both aspects have the potential to minimize leg discrepancies in unipedal balance performance among soccer players at different levels of soccer experience. In addition, methodological discrepancies, such as the testing apparatus used, may also account for our finding of non-significant differences in reach distances between legs. Specifically, force/balance platforms were used in studies [5,18] that reported limb differences in unipedal balance performance, but the YBT-LQ, a device that is more frequently used in the field, was applied in the present study. Thus, one may argue that the YBT-LQ, compared to instrumented biomechanical devices, is not sensitive enough to adequately detect limb differences in unipedal postural control. However, a previous study [6] using the YBT-LQ was able to detect reach distance differences between the legs in young athletes.

Although the observed amount of reach differences between the standing and kicking leg did not reach statistical significance, the value on the YBT-LQ in the ANT direction was above the proposed cut-off value of >4 cm in all four groups and increased with age (from 4.2 to 5.0 cm). When performing the YBT-LQ, anterior reach differences between the legs greater than 4 cm are indicative of an increased risk of sustaining a non-contact lower extremity injury in athletes [7,8]. Even though the increase in limb difference did not reach the level of significance in our study, coaches are nevertheless advised to implement neuromuscular training programs to decrease the risk of lower extremity injuries. Various studies [19,20,21] showed the effectiveness of neuromuscular training regimens (e.g., the FIFA 11+ warm-up program for youth soccer players) to decrease the risk of lower extremity injuries in soccer players, especially those including a combination of balance and strength exercises. Furthermore, Filipa et al. [13] demonstrated that anterior reach distance differences between legs in the YBT-LQ could be reduced after 8 weeks of training (two times per week). The neuromuscular training program was applied in young soccer players (mean age: 15 ± 2 years) and consisted of lower extremity (e.g., single limb hop and hold) and core (e.g., bilateral knee heel) exercises performed on unstable devices (e.g., Airex pad, BOSU ball, Swiss ball).

We acknowledge that this study has some limitations that warrant discussion. Our finding of age but not limb differences in unipedal balance performance is specific to the YBT-LQ, which is a well-established field-based method of investigating dynamic balance. Thus, further research is needed to confirm our results in other types of balance (e.g., static, proactive, and/or reactive balance) and by using additional (e.g., biomechanical) testing methods. Further, the participants included in this study were male sub-elite young soccer players. Therefore, care is needed when generalizing the present findings to other populations (e.g., untrained subjects).

## 5. Conclusions

Older, more experienced soccer players (U19) showed significantly better YBT-LQ performance compared to younger, less experienced ones (U13). Irrespective of age (i.e., years of playing experience), no statistically significant leg differences in balance performance were found. However, limb differences in the ANT direction were above the proposed cut-off value of >4 cm, indicating an increased risk of sustaining a lower extremity injury. Neuromuscular training (i.e., combination of balance and strength exercises) has the potential to decrease the anterior reach distance difference between legs in young soccer players [13]. Because an anterior reach distance difference between the standing and kicking leg greater than 4 cm was already present in the U13 players, we recommend incorporating the YBT-LQ into pre-participation physical examinations for identification of soccer players at risk.

## Figures and Tables

**Table 1:** Characteristics of the soccer players by age.

Characteristic	U13 (*n* = 19)	U15 (*n* = 14)	U17 (*n* = 21)	U19 (*n* = 22)
Age, years	12.0 ± 0.2	13.6 ± 0.5	15.7 ± 0.6	17.1 ± 2.3
Body height, cm	154.7 ± 8.4	171.4 ± 7.7	175.2 ± 7.5	178.9 ± 7.8
Body mass, kg	42.3 ± 8.9	61.4 ± 9.3	67.8 ± 6.8	80.1 ± 8.2
BMI, kg/m^2^	17.7 ± 2.1	20.7 ± 2.2	22.0 ± 2.3	22.0 ± 1.2
Leg length left, cm	83.9 ± 5.5	92.2 ± 3.4	93.2 ± 3.7	91.1 ± 6.4
Leg length right, cm	84.0 ± 5.7	91.9 ± 32	93.0 ± 3.9	90.9 ± 6.2
Leg dominance, left/right	3/16	2/12	3/18	4/18
Training experience, years	7.1 ± 1.0	8.9 ± 1.5	9.8 ± 1.8	10.2 ± 1.9
Training volume, min/week	270	360	360	450

Values are mean ± SD. BMI = Body Mass Index; U13 = under-13 years etc.

**Table 2:** Outcome measures (analysis of variance (ANOVA) with repeated measures on leg).

Measure	U13 (*n* = 19)		U15 (*n* = 14)		U17 (*n* = 21)		U19 (*n* = 22)		*p*-Value (Effect Size)		
	D	ND	D	ND	D	ND	D	ND	Leg	Age	Leg × Age
ANT, % LL	79.1 ± 10.6	80.4 ± 14.0	72.8 ± 7.4	73.3 ± 7.4	77.8 ± 5.5	76.6 ± 6.3	82.4 ± 9.2	80.5 ± 8.2	0.652 (0.05)	0.031 * (0.36)	0.378 (0.21)
PM, % LL	121.1 ± 9.5	119.2 ± 11.3	112.6 ± 8.0	115.3 ± 8.2	119.4 ± 6.9	112.5 ± 4.0	123.0 ± 6.2	124.4 ± 6.0	0.399 (0.10)	0.018 * (0.38)	0.080 (0.31)
PL, % LL	118.9 ± 8.6	121.8 ± 12.1	107.8 ± 9.5	107.0 ± 7.4	114.6 ± 6.0	115.2 ± 9.9	118.7 ± 7.5	119.3 ± 7.3	0.274 (0.13)	0.001 *^,†^ (0.59)	0.383 (0.21)
CS, % LL	106.4 ± 8.4	107.1 ± 11.0	97.8 ± 7.2	98.5 ± 4.3	103.9 ± 4.8	103.0 ± 5.8	108.0 ± 6.7	108.1 ± 6.0	0.751 (0.03)	0.001 *^,†^ (0.53)	0.504 (0.18)

Values are mean ± SD. Figures in brackets are effect sizes (*f*) with 0 ≤ *f* ≤ 0.24 indicating small, 0.25 ≤ *f* ≤ 0.39 medium, and *f* ≥ 0.40 large effects. * significant difference between U15 and U19 players; ^†^ significant difference between U13 and U15 players; ANT = anterior; CS = composite score; D = dominant leg (i.e., kicking leg); LL = leg length; ND = non-dominant leg (i.e., stance leg); PL = posterolateral; PM = posteromedial; U13 = under-13 years etc.
